# A Hybrid Framework for Maritime Surveillance: Detecting Illegal Activities through Vessel Behaviors and Expert Rules Fusion

**DOI:** 10.3390/s24175623

**Published:** 2024-08-30

**Authors:** Vinicius D. do Nascimento, Tiago A. O. Alves, Claudio M. de Farias, Diego Leonel Cadette Dutra

**Affiliations:** 1 Systems Engineering and Computer Science Graduate Program (PESC)/COPPE, Federal University of Rio de Janeiro (UFRJ), Rio de Janeiro 21941-972, RJ, Brazil; cmicelifarias@cos.ufrj.br (C.M.d.F.); ddutra@cos.ufrj.br (D.L.C.D.); 2 Brazilian Navy Research Institute (IPqM), Rio de Janeiro 21931-095, RJ, Brazil; 3 Institute of Mathematics and Statistics (IME), State University of Rio de Janeiro (UERJ), Rio de Janeiro 20550-900, RJ, Brazil; tiago@ime.uerj.br

**Keywords:** maritime surveillance, illegal activities detection, navigation behavior models, expert knowledge integration, active learning

## Abstract

Maritime traffic is essential for global trade but faces significant challenges, including navigation safety, environmental protection, and the prevention of illicit activities. This work presents a framework for detecting illegal activities carried out by vessels, combining navigation behavior detection models with rules based on expert knowledge. Using synthetic and real datasets based on the Automatic Identification System (AIS), we structured our framework into five levels based on the Joint Directors of Laboratories (JDL) model, efficiently integrating data from multiple sources. Activities are classified into four categories: illegal fishing, suspicious activity, anomalous activity, and normal activity. To address the issue of a lack of labels and integrate data-driven detection with expert knowledge, we employed a stack ensemble model along with active learning. The results showed that the framework was highly effective, achieving 99% accuracy in detecting illegal fishing and 92% in detecting suspicious activities. Furthermore, it drastically reduced the need for manual checks by specialists, transforming experts’ tacit knowledge into explicit knowledge through the models and allowing continuous updates of maritime domain rules. This work significantly contributes to maritime surveillance, offering a scalable and efficient solution for detecting illegal activities in the maritime domain.

## 1. Introduction

Maritime traffic plays a crucial role in global connectivity and international trade, serving as a vital artery for the world’s economy. Millions of tons of goods are transported across the oceans every year, driving economic growth and development in coastal countries [1]. The strategic importance of maritime transport is undeniable, as it is responsible for over 90% of global trade [2]. Additionally, the coastal areas of some countries are an essential source of employment and revenue for many nations, sustaining coastal communities and promoting socioeconomic development.

However, this complex network of maritime activities also presents significant challenges, such as the need to ensure the security of maritime routes, the protection of the marine environment, and the prevention of illicit activities [3]. Therefore, effective monitoring of the maritime domain by local maritime authorities (MAs) becomes essential to ensuring the efficiency, safety, and sustainability of maritime operations worldwide.

Through Maritime Domain Awareness (MDA), MAs can monitor their Exclusive Economic Zones (EEZs) to ensure safe maritime traffic and the economic exploitation of the coast by the country [4]. To improve maritime traffic security, it is essential to strengthen intelligence processes in maritime surveillance. Detecting illegal activities carried out by vessels is crucial for enhancing navigation safety, as well as improving the efficiency and protection of maritime operations [3]. The detection of navigation behaviors such as fishing trajectories, loitering, spoofing, encounters (or rendezvous), and dark ships is essential for situational awareness and, consequently, for the detection of illegal activities [5].

Several works in the literature have addressed how to detect individual vessel behaviors [6,7,8,9], mainly anomalies. In [10], the relationship between these navigation behaviors and the activities performed by the vessels was addressed, and how it is possible to detect activities through this relationship. Vessel behaviors can be observed through sensors, while the activities performed are part of the human will for which the vessels are used. In a literature review on the detection of anomalous vessel behaviors conducted in [11], it was concluded that specialized knowledge is an important aid in understanding ship navigation behavior, although experts with knowledge in the maritime area are mixed and diverse, making it difficult to form a unified pattern. Thus, establishing a data-driven method incorporating specialized knowledge is an important research direction.

A significant portion of the literature on maritime surveillance mostly concentrates on anomaly identification, as evidenced by the works of [5,6,11,12]. There is a scarcity of research on the identification and detection of illegal activities in the maritime domain. As an illustration, Ref. [10] discussed activity classification, focusing solely on the ontological viewpoint. In [13], the authors introduced a method to infer illegal maritime activities in real time by examining the multiscale spatial behavior of entire fleets, specifically groups of vessels that transmit their location via AIS. However, in our investigation, we did not find any work attempting to detect illegal vessel activities using a methodology similar to ours. Hence, the objective of this study is to address this gap by presenting a framework capable of detecting illegal activities carried out by vessels through the fusion of vessel behavior detection models with rule outputs created based on expert knowledge.

Many maritime domain monitoring systems used by maritime authorities are based on kinematic rules created by experts. Rule-based approaches aim to detect a restricted set of vessel activities, areas, or dynamic characteristics of vessel trajectories or trajectory evolutions through predefined rules and limits. Automated Behavior Monitoring (ABM), developed by the European Maritime Safety Agency (EMSA) [14], enhances maritime surveillance by incorporating a collection of predefined rule-based scenarios. These scenarios enable the automatic activation of detection for vessels arriving in specific areas, following unconventional routes, or being located in designated areas.

However, a significant issue with these systems is the creation of thousands of rules and the triggering of a large number of false alerts, which can overwhelm operators. In this context, our framework seeks to enhance this activity detection process by reducing the necessary interventions from specialists, ensuring that they only intervene in vessel trajectories that truly require attention. Therefore, our framework aims to achieve the following primary objectives:

- Detect illegal activities in the maritime domain involving the use of vessels;
- Reduce the number of trajectories that specialists need to analyze;
- Integrate the tacit knowledge of specialists with data-driven methods;
- Convert the tacit knowledge of specialists into explicit knowledge through machine learning models;
- Ensure the scalability of the framework for detecting other activities, behaviors, and rules.

To reach these objectives and show that our framework works for detecting illegal activities on vessels, we will use a combination of methods in a JDL framework, using both synthetic and real datasets for training and testing.

## 2. Materials and Methods

In this section, we will present the methodology adopted in this work, along with a brief discussion of essential concepts, including ensemble models, active learning, and the JDL framework. We will detail the framework’s implementation, navigation behaviors, rules defined based on expert knowledge, and active learning modeling. Additionally, we will describe the training and testing data and experiment design. Finally, we will present the main results achieved.

**Figure 1:**
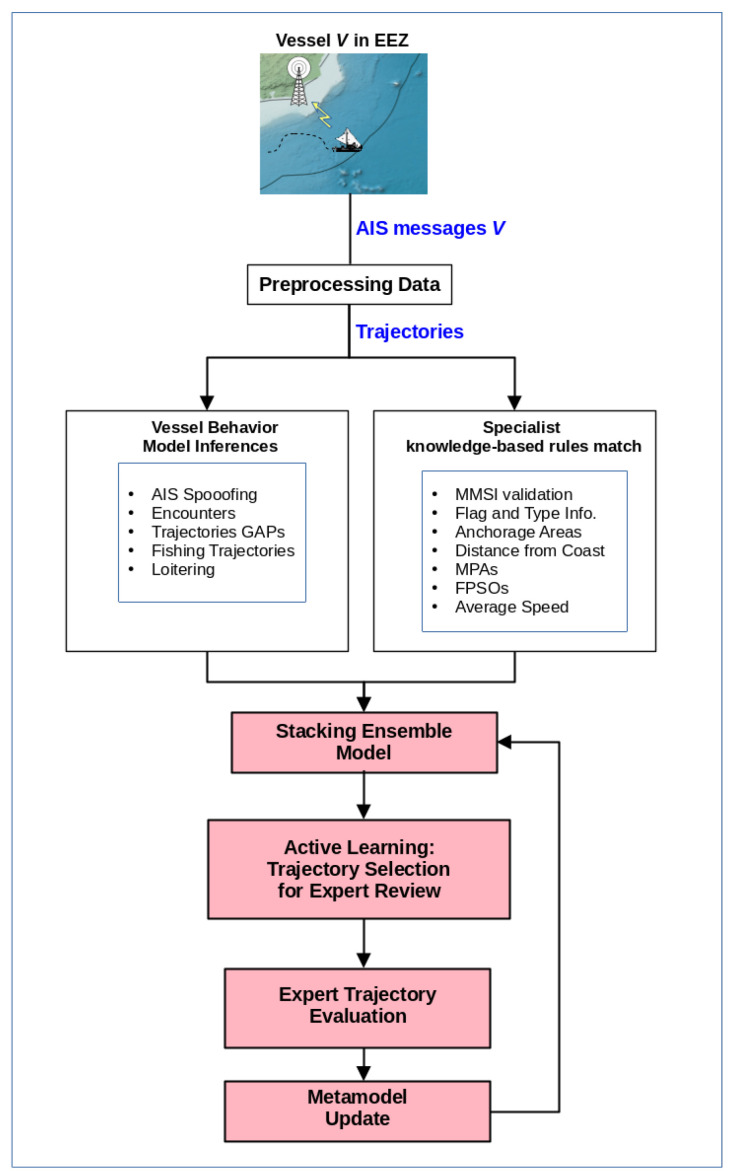
A summarized process for detecting illegal activities in the maritime domain.

To provide a brief overview of the detection process, Figure 1 shows a summarized view of how our framework for detecting illegal activities operates. First, trajectories are created from AIS data received from data sources. Next, these trajectories are applied to behavior models as well as to rules defined based on expert knowledge. The outputs are then combined into a stacking ensemble model, and active learning is used to select the trajectories with the highest uncertainty. Finally, the expert can evaluate the trajectory and classify it, allowing the model to be updated accordingly. In the following sections, we will provide more details about each part of the process and how the model training is conducted.

### 2.1. Trajectory Extraction

**Figure 2:**
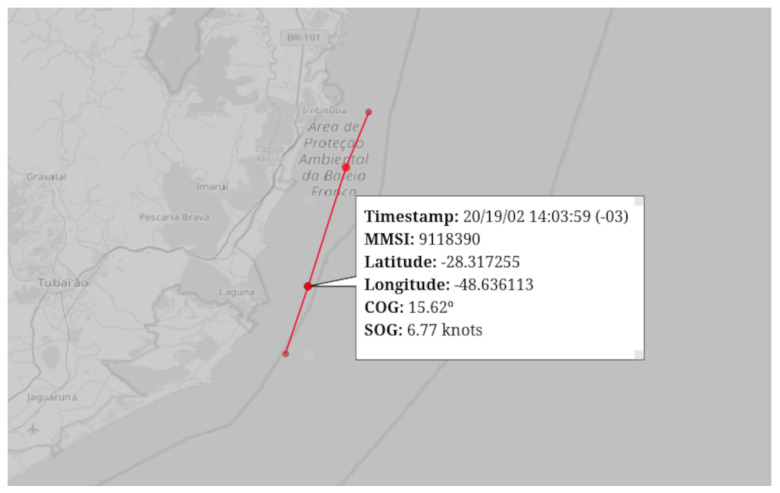
Vessel trajectory example.

A trajectory is a sequence of points in space and time that describes the movement of a vessel. Each point in the trajectory is characterized by spatial coordinates (latitude and longitude) and a timestamp. The trajectory provides a detailed view of the vessel’s path over time. Additionally, we have speed over ground (SOG) and course over ground (COG) data, which indicate the vessel’s speed and direction relative to the sea floor, as illustrated in Figure 2. A normal trajectory follows an expected movement pattern based on historical behaviors or established rules. For example, for fuel economy reasons, cargo vessels usually follow a predefined commercial route at a constant speed and without abrupt deviations. In the case of fishing vessels, they may exhibit characteristic fishing trajectories in the locations where they are actually fishing. An anomalous trajectory, on the other hand, may show significant deviations from the expected pattern, indicating atypical or suspicious behaviors. The next section discusses these behaviors and how to spot them.

### 2.2. Vessel Behaviour Modeling

Vessel behaviors are the observable responses of a vessel to external and internal stimuli, which are not necessarily associated with a specific objective. These behaviors can be routine, such as following a specific trade route, or they can be indicative of specific activities, both legal and illicit. Understanding and detecting these behaviors is crucial for maritime safety, security, and surveillance.

The detection and monitoring of vessel behaviors primarily rely on various sensors. The most prominent among these is the AIS (Automatic Identification System), which is a tracking system most commonly used on ships and by vessel traffic services. AIS transponders automatically broadcast information, such as their position, speed, and navigational status, at regular intervals via a VHF transmitter. Other sensors include radars, which detect and map ships’ positions based on reflected radio waves, and satellite imagery, which provides a view of maritime regions and can capture vessel movements.

Several behaviors have been identified and studied in maritime literature. In this study, we will include the most common behaviors, with a brief explanation of each in the next subsections. We have implemented each behavior model below, and these take vessel trajectories as input and outputs a float value representing the probability of the detected behavior. In the following subsections, we will provide brief explanations of each behavior and rule implemented in our framework.

#### 2.2.1. AIS Spoofing

**Figure 3:**
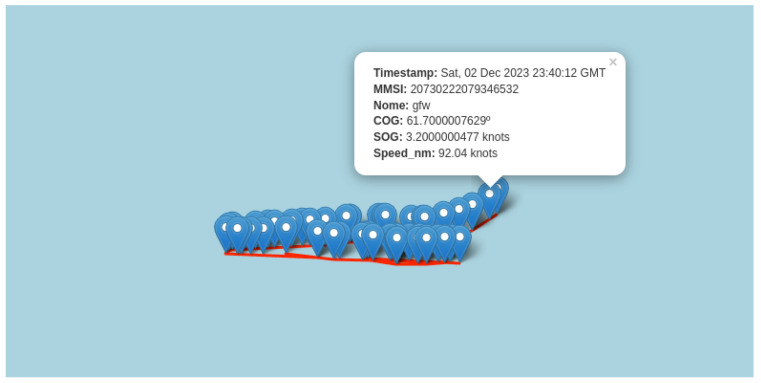
An instance of spoofing. The SOG given by the AIS is 3.2 knots. However, when we compute the speed using the coordinates and the time taken to move between them, we obtain a speed of 92 knots.

The malicious alteration of AIS data transmitted by vessels themselves to disguise their identity or location, known as spoofing, has been demonstrated as possible in various studies [15,16]. This practice can mask or facilitate illegal activities, disrupt monitoring systems, and create navigation risks. Some studies have attempted to detect AIS spoofing, such as [17,18,19]. In [17], an architecture was proposed to detect AIS data spoofing in an online data stream. The architecture stores the vessel’s MMSI in a hash, and for each associated trajectory it computes the haversine distance between consecutive points and the average speed. If the average speed exceeds 50 knots, the trajectory is considered spoofed. Figure 3 shows an example of spoofing. The vessel’s AIS reports an SOG of 3.2 knots, but when we calculate the speed based on latitude, longitude, and timestamp positions we obtain a speed of 92 knots.

Thus, to detect AIS spoofing behavior, we calculate the speed in knots between each consecutive points of the trajectory. If at any point the speed exceeds 50 knots, it will be considered an impossible speed, and the trajectory will be identified as a spoofing case. Additionally, trajectories with points in impossible locations, such as crossing land, will also be considered spoofed.

#### 2.2.2. Encounters at Sea

In the context of maritime activities, encounter refers to an unplanned or accidental meeting between vessels, often happening by chance without prior arrangement. In contrast, rendezvous denotes a planned and intentional meeting at a specific time and location, involving prior coordination and agreement between the involved parties. While an encounter is spontaneous and can occur unexpectedly, a rendezvous is deliberate and organized, commonly used for illicit activities or specific purposes where vessels need to meet at a predetermined spot. To detect a rendezvous, first we need to detect the encounter. To detect the intention of an encounter, we need to analyze the context behind the situation. Hence, for this specific vessel behavior, first we will be concerned with detecting the encounter.

**Figure 4:**
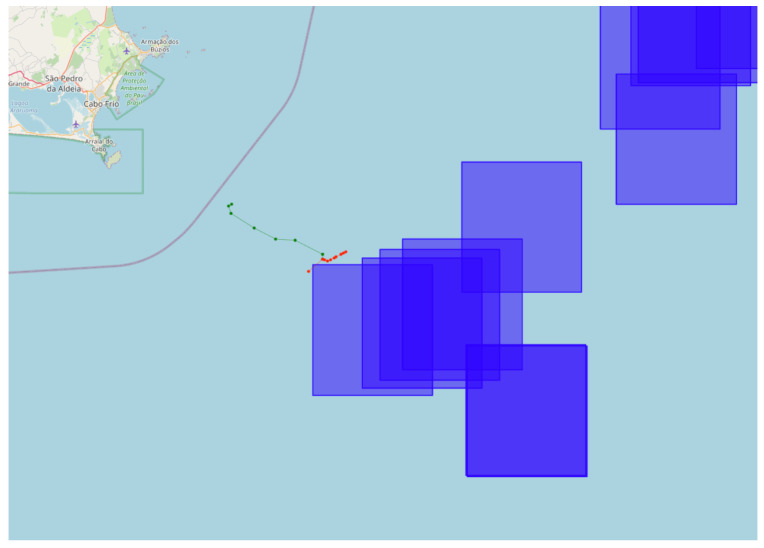
An example of an encounter at sea between vessels. The green points represent the trajectory of Vessel 1, while the red points represent Vessel 2. The blue squares represent FPSO areas. The image shows that the vessel had an encounter outside the FPSO area.

Two or more ships having an encounter in open waters may be involved in the transfer of goods or people. This can be indicative of trade but also of activities such as smuggling. While an encounter can be considered suspicious behavior, that is not always the case. In oil exploration areas, many support vessels transport personnel, oil, and support materials. In these areas, it is common to practice ship-to-ship transfer, which is the transfer of cargo between vessels at sea. In these situations, the proximity between vessels is considered normal, as the vessels are near ports where they can moor close to each other. Figure 4 shows an example of an encounter between vessels. In green, we have vessel 1; in red, we have vessel 2; and the blue squares represent oil exploration areas.

Some studies, such as [20,21,22,23], have proposed solutions for detecting these encounters, taking into account their intent. However, for simplicity, in this work, we will use only AIS positions and timestamps to detect vessels that have positioned themselves less than 200 m apart. To do this, we use a geohash-based coding system. Geohash is a coding system that transforms geographic coordinates (latitude and longitude) into an alphanumeric string [24]. Geohash’s main feature is that it divides the geographic space into a hierarchical grid of square cells of different sizes, increasing the location’s precision with each additional character in the string.

The H3 library [25] is a Python implementation of geohash. Uber developed the H3 library to divide the world into a hierarchical hexagonal grid. H3 integrates geohashing concepts with the flexibility and efficiency of hexagonal grids, a preferred choice due to several favorable geometric properties over square grids. Therefore, for encounter detection in this work, we group the hashes generated by each AIS position using resolution 9 (200 m) and use a time window of 4 h. Thus, for one or more vessels to be considered in an encounter, they must be in the same geohash cell within a 4 h window and more than 10 nautical miles from the coast. The choice of resolution 9 was based on the need for spatial precision that allows capturing relevant encounters between vessels without generating an excessive number of data. We selected the 4-hour window to detect proximity, even if both vessels take up to 2 h to emit an AIS signal.

#### 2.2.3. Dark Ships

Vessels that turn off their AIS transponders to avoid detection often indicate an intent to engage in illegal activities. Sometimes, this practice can lead to confusion when the vessel navigates through areas with signal interference or bad weather. Therefore, these areas need to be known so that we can accurately infer this type of behavior.

**Figure 5:**
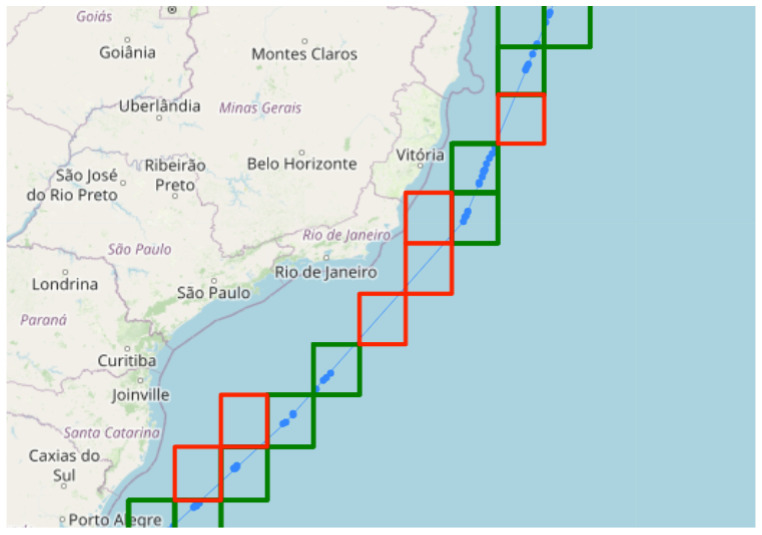
The image shows an example of gap detection in a vessel’s trajectory. The blue points reflect the vessel’s trajectory. The red squares indicate where the vessel should have transmitted the AIS signal, while the green squares represent where the vessel did transmit. The red squares represent transmission gaps.

In the literature, we found a variety of attempts to solve this problem, mainly by using active sensors for detection. For non-collaborative systems, works such as [26,27] used radars, which, when fused with AIS, have both AIS and radar trajectories associated, and an anomaly can be detected if they diverge. Other works attempted to solve the problem using only the AIS sensor [28,29]. However, using only the AIS sensor, we can detect transmission gaps in the trajectory and assume that the vessel may have turned off the AIS transponder. Figure 5 shows an example of trajectory gaps. The blue points represent where the vessel transmitted the AIS signal. The green squares represent regions where the vessel transmitted within the area, while the red squares indicate transmission gaps, meaning the vessel should have transmitted within that region.

To calculate these gaps in the trajectories, we first create a unique trajectory for each vessel, regardless of whether it remains stationary or not. Next, we generate a geohash with a precision of 3 (156 km). If we consider that a vessel transmits an AIS signal at least every 2 h and extrapolate its speed to 40 knots, then the vessel should emit a signal every 80 NM (approximately 148 km). Thus, by mapping all the geohash squares that the vessel passed through, as shown in Figure 5, we have in red all the squares where the vessel’s trajectory crossed but did not transmit.

Another important factor to verify is whether the area where the vessel should have transmitted has any electromagnetic interference problems. To do this, we store a history of geohashes with the respective quantities of transmissions already made from that region. When we identify a gap in a vessel’s trajectory, we verify if AIS transmissions have previously taken place in the red regions. If there have been no transmissions in that region, it would not be considered a gap in the trajectory. Therefore, in our work, we consider these transmission gaps along with the geohashes’ transmission histories to determine whether the vessel should have transmitted an AIS signal from that position or not.

#### 2.2.4. Fishing Trajectories

**Figure 6:**
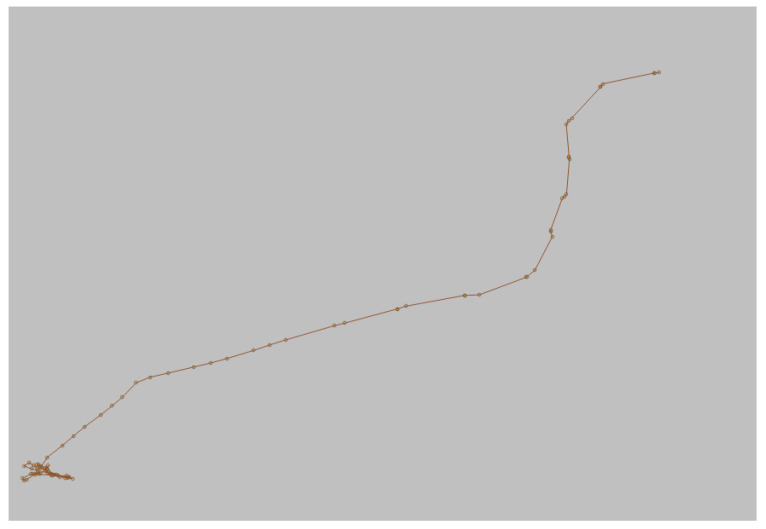
A fishing trajectory example.

Fishing vessels in normal movement from one region to another typically behave like cargo vessels, trying to follow an optimized trajectory. However, when a ship arrives at fishing spots and starts its target activity, it needs to perform specific maneuvers. For each type of fishing activity, there are characteristic maneuvers of the vessel, as exemplified in Figure 6. Movement patterns reveal a vessel’s fishing activity, which allows for monitoring in restricted areas. The detection of fishing trajectories has been addressed by several works using AIS sensor data in machine learning models [30,31,32,33,34,35,36]. In this work, we use a similar solution proposed by [32,33,36], where a recurrent neural network (RNN) is used on the time series of the trajectory. The dimensions used in the RNN model are speed, angular difference between points, time difference, distance difference, and acceleration.

Given the latitude and longitude of these points, we can use the haversine distance to calculate the distance between two points on the surface of a sphere. Therefore, the distance in nautical miles can be calculated using Formula (1).

(1) $$d=2r\cdot \arcsin\left(\sqrt{\sin^{2}\left(\frac{\varphi_{2}-\varphi_{1}}{2}\right)+\cos\left(\varphi_{1}\right)\cos\left(\varphi_{2}\right)\sin^{2}\left(\frac{\lambda_{2}-\lambda_{1}}{2}\right)}\right)$$

where:

- *d* is the distance between the two points, in nautical miles.
- *r* is the radius of the Earth, approximately 3440 nautical miles.
- $\varphi_{1}$ and $\varphi_{2}$ are the latitudes of the two points, in radians.
- $\lambda_{1}$ and $\lambda_{2}$ are the longitudes of the two points, in radians.

In terms of the time difference between two points, we use the unit of minutes. To calculate the speed and acceleration in knots, we simply divide the distance and speed, respectively, by the time in hours. Finally, to calculate the angular difference between the points, we can use Formula (2).

(2) $$\Delta \theta =\arctan2(\sin\left(\theta_{1}-\theta_{2}\right),\cos\left(\theta_{1}-\theta_{2}\right))$$

where $\theta_{1}$ and $\theta_{2}$ are the directions at each point, and arctan2 is a variation of the arc tangent function that takes into account the sign of both the sine and cosine components to determine the quadrant of the resulting angle. This same strategy for detecting fishing trajectories was used in [36].

After creating the RNN model for detecting fishing trajectories, we trained it using the Global Fishing Watch (GFW) fishing trajectories dataset [37]. Thus, the model infers a probability value from 0 to 1, where 1 indicates 100% likelihood of a fishing trajectory and 0 indicates otherwise.

#### 2.2.5. Loitering

Loitering involves a ship or boat spending an abnormally long time in the same area or performing slow movements around a larger area without displacing itself a significant distance. Vessels that remain stationary or move in circles indicate possibly waiting for another ship or engaging in activities like illegal fishing.

Several studies on loitering detection have generally described it as detecting an object at a scene for a period of time that exceeds a given threshold [38,39,40]. However, to detect a loitering trajectory, we innovatively created a method using an RNN model, similar to the detection of fishing trajectories. First, using the MarineCadastre dataset [41] with AIS data from the coast of the United States, we detected vessel encounters. For vessels to meet, they first need to slow down to a complete stop. This deceleration and subsequent low-speed movement for a few hours in a location are characteristic of loitering trajectories that can precede an encounter between vessels. By detecting the trajectories that performed these encounters, we extracted these trajectories to train our RNN model. Then, we created an RNN architecture with the same dimensions that we used to detect fishing behaviors (speed, distance difference, time difference, angular difference, and acceleration). Finally, we trained the model with the trajectories extracted from the encounters in the MarineCadastre dataset.

#### 2.2.6. Anomalous Navigation

Deviations from typical navigation patterns could signify a vessel’s loss, technical issues, or involvement in illicit activities. Most studies in the literature treat anomalies in vessel routes in a binary manner, checking the most common routes and considering trajectories that deviate from these patterns as anomalous [42,43,44,45].

In the present study, instead of monitoring a specific area, we observe the entire Brazilian coast. Therefore, it will be necessary to detect the normal pattern of each navigation channel along the coast. To simplify, we will not use the concept of anomaly as a behavior, but rather as an activity.

Some combinations of behaviors described earlier, when associated with certain rules defined by experts, can be classified as anomalous activities. For instance, we can classify a vessel navigating with an invalid MMSI and exhibiting spoofing behavior as engaging in an anomalous activity. As a result, we will adopt an anomalous activity class for situations where a combination of behaviors and rules is judged to be anomalous by experts.

### 2.3. Defining Knowledge-Based Rules

**Figure 7:**
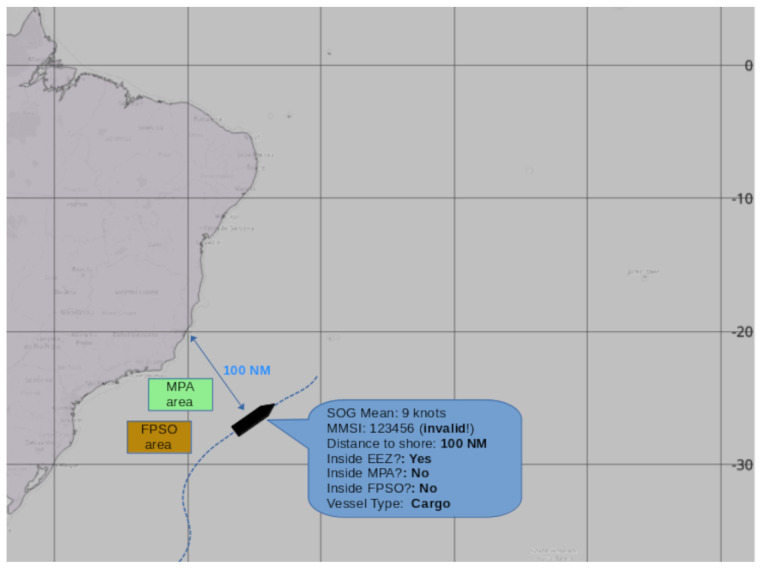
In the picture, we can see rules established by the expert based on his expertise. For example, the expert establishes kinematic rules for specific areas (e.g., MPA and FPSO areas), vessel identification characteristics, the distance from the shore where certain activities occur, and so on.

In various maritime domain monitoring systems, it is common to use sets of knowledge-based rules developed by experts in the field. These rules are formulated based on years of experience and deep knowledge of navigation patterns, expected vessel behaviors, indicators of suspicious activities, and laws. Knowledge-based rules are typically created to identify deviations from normal maritime operation patterns. They cover a variety of criteria, such as unexpected trajectories, anomalous speed variations, the use of invalid identifiers (MMSI), and behaviors indicative of spoofing or illicit operations. Kinematic behaviors refer to a vessel’s movement pattern, including aspects such as speed, direction, and any changes in its course or speed over time [46]. Some agencies, such as the European Maritime Safety Agency (EMSA) [14], have models called Automated Behavior Monitoring (ABM). Examples of behaviors can be seen in Figure 7. The figure shows some rules created by experts, such as entry into MPA and FPSO areas, validity of the MMSI number, entry into the EEZ, etc. The goal of ABM is to automatically detect and highlight vessel behaviors that may be of interest or concern. However, excessive use of such rules can become impractical because specialists need to verify each rule trigger. Therefore, in maritime monitoring systems, it is simple to create thousands of kinematic rules, with the operator responsible for verifying each trigger and updating these rules.

However, when combined with other navigation behaviors, these rules can become important tools in detecting illegal activities. For instance, an expert can identify prohibited fishing areas and create polygons to delineate them. If a fishing vessel enters these areas and exhibits a fishing trajectory, it can be inferred that illegal fishing activity is taking place.

In this study, we will apply the expert-derived rules listed in Table 1 to each trajectory.

These rules apply to all trajectories and normalize the outputs. We convert the values of binary responses to either 1 or 0. Finally, we apply these rules to the trajectories and insert them into the SQLite database for later use in the metamodel and active learning.

### 2.4. Defining and Selecting Vessels’ Activities

Maritime Authorities (MAs) are typically responsible for a variety of activities in their jurisdictional waters, including coastal monitoring, protection of human life at sea, law enforcement, protection of MPAs, protection of critical infrastructure, and response to environmental crimes [48]. Large-scale solutions are necessary due to the large number of activities that require monitoring, as human agents cannot effectively perform these tasks across the entire Exclusive Economic Zone (EEZ). For example, Brazil’s EEZ is the tenth largest in the world, with 3.5 million square kilometers [49], and, consequently, the use of sensors and automated applications to alert human operators has become essential.

MAs worldwide are concerned about illegal activities carried out by vessels in their maritime domain. Some examples include illegal fishing, protection of marine protected areas (MPAs), protection of critical infrastructure, combating trafficking (arms, drugs, humans, and money), and navigation safety situations. However, automating the identification of these activities presents a significant challenge, as it requires not only the identification of the vessel’s behavior, but also the identification of the human intent (commander, crew, owner, etc.) behind the vessel’s use.

In [10], an ontology-based approach was proposed to detect vessel activity based on vessel behavior. The author argues that modeling and exploring uncertain information is a fundamental enabler for recognizing vessel activity, given the high variability of vessel behavior and the inherent uncertainty of its intent. As a result, vessel behavior is part of the observed activity. In this work, we consider the detected vessel behaviors in conjunction with the expert-created rules to detect vessel activity.

Detecting all these activities would be an unfeasible scope for this work, both due to the lack of historical data and sensors and due to the broad scope. Therefore, we will focus on activities where academic data exist so that we can train our models and present tests demonstrating that our framework works and can be scalable. Therefore, this work will classify activities into the following categories:

- *Illegal fishing:* fishing is considered illegal when a vessel engages in fishing within an area where fishing is not allowed, or when vessels fish within the EEZ of a foreign country [50];
- *Suspicious activity:* an activity that, according to the expert’s judgment, might be conducted to commit an illicit activity. However, some evidence is missing (due to uncertainty in certain data) to confirm this specific activity (such as illegal fishing, trafficking, etc.). Based on the expert’s experience, these activities should be considered with a higher level of severity than a typical anomalous activity. For instance, encounters between ships situated across a distance of 12 nautical miles from the shore means they may be engaging in the transportation of goods or the transfer of arms. Another example would be unidentified vessels exhibiting fishing patterns between 180 and 200 nautical miles, which is often indicative of illegal fishing activity;
- *Anomalous activity:* an expert classifies an activity as anomalous when a specific set of behaviors and/or rules appear uncommon but do not immediately suggest illegal activity. For instance, if a vessel is performing atypical maneuvers in a region that is usually devoid of activity, the expert may classify it as anomalous. However, in this situation, there is no evidence of illicit conduct, just a deviation from the norm;
- *Normal activity:* when a navigation activity is considered normal for that locality.

At first, there is no association between vessel behaviors and activities. This correlation exists empirically in the expert’s mind and will be established through the expert’s evaluation of the trajectories. In the following sections, we will outline the strategy for selecting trajectories for the expert to evaluate so that this correlation can be developed. In this work, we will use these four activities as the primary labels for classification. In the Section 2.8, we will define objective criteria for the expert to classify each activity.

### 2.5. Implementation of Ensemble Models and Stacking Strategy

Ensemble models are a machine learning technique that involves combining several models to increase accuracy and performance in solving complex problems. The central idea behind ensemble models is that, by combining multiple models’ predictions, it is possible to obtain more accurate and robust results than any single model could provide. Historically, the concept of ensemble models began to gain prominence in the 1990s with the development of algorithms such as bagging and boosting. These methods demonstrated that aggregating multiple learning models, each contributing its own perspective or experience, can lead to significantly improved performance, particularly in complex classification and regression tasks. The basic premise is that, while a single model may have its limitations and be subject to specific errors, the combination of multiple models can compensate for these weaknesses, resulting in greater accuracy and reliability in predictions [51]. As one of our objectives is to reduce uncertainties when classifying activities, this strategy will help us by allowing the strengths of each behavior model to compensate for the weaknesses of the others, generating predictions with lower uncertainty.

Our primary strategy involves employing an ensemble model technique known as stacking [52]. Stacking is an ensemble learning method that combines the predictions of several different machine learning models to enhance overall predictive accuracy. The stacking process can be divided into two main phases:

1. **Training of base models:** multiple models, referred to as base learners, are trained using the training dataset. These models can be of various types, such as logistic regression, decision trees, random forests, neural networks, etc. Each base learner captures a different aspect of the patterns within the data.
2. **Prediction combination:** after training, the predictions from the base models are combined to form a new set of features. This feature set is then used to train a metamodel (*meta-learner*), which is responsible for learning the optimal way to combine the predictions of the base models to produce the final prediction. The metamodel can be, for instance, a linear regression, gradient boosting, or any other supervised learning model.

Mathematically, let $D=\{\left(x_{1},y_{1}\right),\left(x_{2},y_{2}\right),\ldots ,\left(x_{n},y_{n}\right)\}$ be the training dataset, where $x_{i}$ are the input features and $y_{i}$ are the corresponding outputs. Consider $M_{1},M_{2},\ldots ,M_{k}$ as the base models, and $M_{meta}$ as the metamodel.

First, each base model, $M_{j}$, is trained on *D* to learn the function $f_{j}:x_{i}\to {\hat{y}}_{ij}$, where ${\hat{y}}_{ij}$ is the prediction of model $M_{j}$ for input $x_{i}$. Next, the predictions of all base models are combined to form a new feature vector, ${\hat{x}}_{i}=\left({\hat{y}}_{i1},{\hat{y}}_{i2},\ldots ,{\hat{y}}_{ik}\right)$.

Finally, the metamodel, $M_{meta}$, is trained to learn the function $f_{meta}:{\hat{x}}_{i}\to {\hat{y}}_{i}$, where ${\hat{y}}_{i}$ is the final combined prediction.

(3) $${\hat{y}}_{i}=f_{meta}\left({\hat{y}}_{i1},{\hat{y}}_{i2},\ldots ,{\hat{y}}_{ik}\right)$$

This process allows stacking to leverage the strengths of different learning models, which can result in a significant improvement in predictive performance. The effectiveness of stacking is particularly notable in scenarios where there is significant heterogeneity among the base models. This diversity allows the metamodel to develop an enhanced ability to recognize the most efficient way to integrate individual predictions from the base models, resulting in superior accuracy in the final prediction. As a metamodel, we will use the random forest model for its robust ability to reduce overfitting and improve prediction accuracy. The random forest, which is an ensemble of multiple decision trees, combines the predictability of different base models and captures various nuances in the data. Its non-parametric nature allows it to efficiently handle high-dimensional and heterogeneous data, common in the detection of illegal vessel activities.

In our case, we will use the navigation behaviors presented in Section 2.2 and the expert-created rules in Section 2.3 as base models. We will use the output of these models as input for the metamodel, specifically the random forest model. However, even combining behavior models with expert rules, we will still face the problem of a lack of labels for activities. In the next section, where we discuss active learning, we will address this problem in more detail.

### 2.6. Active Learning for Efficient Data Labeling

Significant advances in data collection and storage technology allow for the accumulation of large numbers of data in a wide range of real-world applications. Labeling these data, on the other hand, is expensive because it requires human effort and expertise. Semi-supervised learning is concerned with methods to improve learning performance by using unlabeled data in addition to automatically labeled data. The basic idea behind semi-supervised learning is that, by combining a small number of labeled data with a large number of unlabeled data, the model can learn to make better predictions than it would using only labeled data [52,53].

Active learning is a process in which semi-supervised methods are combined with human expert intervention. In active learning, the machine learning model selects the most informative examples from the unlabeled dataset and asks a human expert to label them. This is especially useful when data labeling is expensive or time-consuming. In an active learning scenario, the machine learning algorithm not only learns from previously labeled data but also has the ability to request labels for specific examples from the unlabeled dataset. The idea is that the model will improve more quickly if it can obtain labels for the examples it finds most uncertain or informative. To make this efficient, active learning algorithms typically use some type of query strategy. They can, for example, select examples where the model’s predictions are most uncertain, or examples that are on the decision boundary between two classes. When we have a large unlabeled dataset and a human expert who can provide accurate labels, but the expert’s time is limited, active learning can be very effective [52].

Given its unique characteristics, our problem presents an exceptional opportunity for the application of active learning. First, we start with unlabeled data, and the expert needs to perform a “cold start” on the labels to provide context. This is a common scenario in many real-world applications where data collection is abundant but labeling is expensive due to the need for human expertise. After the cold start performed by the expert, active learning can be trained and begin querying the user for labels. The model will select data that has the highest probability of increasing its prediction accuracy. Thus, with each label provided by the user, the model’s weights are updated, and, consequently, the model’s accuracy increases. In our case, the model used in active learning is the random forest. In the next section, we will present how our JDL-based framework is organized, from data acquisition to activity prediction.

### 2.7. A Framework for Vessel Illegal Activities Classification

The Joint Directors of Laboratories (JDL) Data Fusion Model serves as a conceptual framework that directs the integration and fusion of data from various sources. This model was introduced by the Joint Directors of Laboratories, a research and development group of the United States Department of Defense, to standardize and improve data fusion processes in surveillance and defense systems [54].

**Figure 8:**
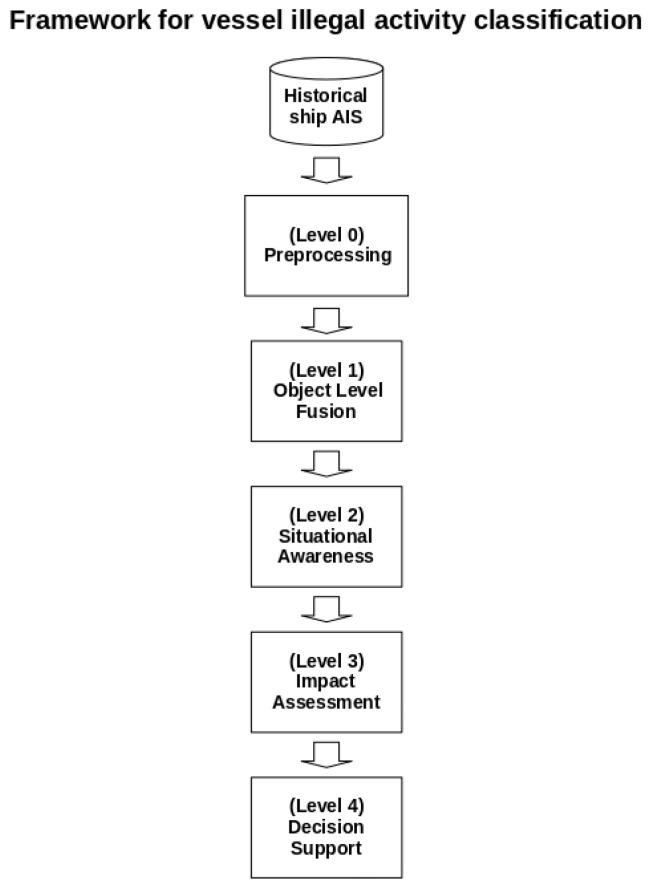
Levels of the JDL-based framework process.

Since our problem involves the use of sensors, fusion of behavior models, and expert-created rules, a certain complexity arises that requires the formal use of a framework to organize our knowledge. We based our approach on the JDL model due to its ability to provide a structured and hierarchical approach to data fusion. This model efficiently integrates information from multiple sources, resulting in a more comprehensive and accurate situational picture. In contexts where critical decisions rely on data collected from various sensors and systems, this feature is crucial. Thus, our framework presents five levels: preprocessing, object-level fusion, situational awareness, impact assessment, and decision support. Figure 8 shows the levels of the framework based on JDL. In the following sections, we will present details about each level of our framework.

#### 2.7.1. Preprocessing

Data preprocessing is a crucial step to ensure the quality and integrity of subsequent analyses. Data can be obtained from different sources and sensors, such as AIS, radars, satellite images, etc. The data used in this study come from raw AIS data. Each row in the dataset comprises latitude, longitude, speed over ground (SOG), course over ground (COG), MMSI, vessel name, and a timestamp. As a result, the primary preprocessing steps are:

- Loading raw AIS data: the raw AIS data are initially loaded directly from the available files using the Python Pandas library;
- Cleaning missing or invalid data: after loading, rows with missing or incorrect data are cleaned. A trajectory must consist of at least five points, so missing cases are removed;
- Transforming AIS data into geodataframes: the cleaned data are then transformed into geodataframes using the geopandas library [55]. This transformation allows for more efficient and accurate manipulation of geospatial information, facilitating data analysis and visualization;
- Converting to trajectories with MovingPandas: finally, the geodataframes are converted into trajectories using the MovingPandas library [56]. This conversion is essential for manipulating the vessels’ movements temporally and spatially, allowing for detailed analyses of their trajectories and navigation behaviors.

This preprocessing makes sure that the input for subsequent processes is in the form of geopandas trajectories and geodataframes. Finally, we store the trajectory data as serialized objects in an SQLite database to facilitate access. This storage format is efficient and allows for quick and flexible queries. We convert each trajectory into an object format, encapsulating all points and associated information, serialize it, and store it in the database.

#### 2.7.2. Object Level Fusion

At this level, we receive the vessel trajectories as input and apply the models for various behavior detections from Section 2.2, as well as the expert rules from Section 2.3. If another sensor type, such as radar, were in use, we could perform object-level fusion by integrating AIS data with radar data for the same contact.

Here, we instantiate the classes used to detect each behavior and rule. These classes utilize the trajectory lists constructed during preprocessing as their parameters. After processing the trajectories, the outputs are stored in a dataframe, which will later be used in the metamodel.

This is one of the most processing-intensive parts of the framework, as the trajectories are not only processed by pre-loaded models but also by algorithms that examine each point of the trajectory, such as detecting proximity between trajectories and identifying gaps. Hence, at this framework level, the outputs from each behavior detection model and the expert-created rules serve as distinct data sources, which we will correlate at the next level.

#### 2.7.3. Situational Awareness

This level is responsible for combining the outputs of navigation behaviors and expert rules, providing a global context for the trajectory situation. This level is also responsible for storing the data in a database, preparing the data for use in active learning, and presenting the data intuitively to the user.

To combine the data, we first create a dataframe where each row contains columns with data on the outputs of each model and expert rule, correlated with the respective trajectory. Then, these data are stored in a database (SQLite). Using a database facilitates manipulation and speeds up the loading of data for model execution. Once completed, the next level can utilize these data as input for its execution.

Practically, a class called *Situational Awareness* is instantiated, and an *Object Level Fusion* is provided as an argument in the constructor. Next, the fuse method is called to merge all behaviors, centralize all data associated with each trajectory, and make them ready for the subsequent stage.

#### 2.7.4. Impact Assessment

The impact assessment level focuses on data interpretation by the user, training/updating the metamodel, and predicting activities. First, this level is responsible for creating active learning and the expert’s cold start. The metamodel data are loaded from the database. If there are not at least 100 labels provided by the expert, the user interface for the cold start is used, selecting 100 trajectories randomly and presenting them to the user for labeling. Once we reach 100 labeled trajectories, we use the active learning user interface to train the random forest model with the labeled data. The model then selects trajectories that have the highest probability of increasing the system’s accuracy.

**Figure 9:**
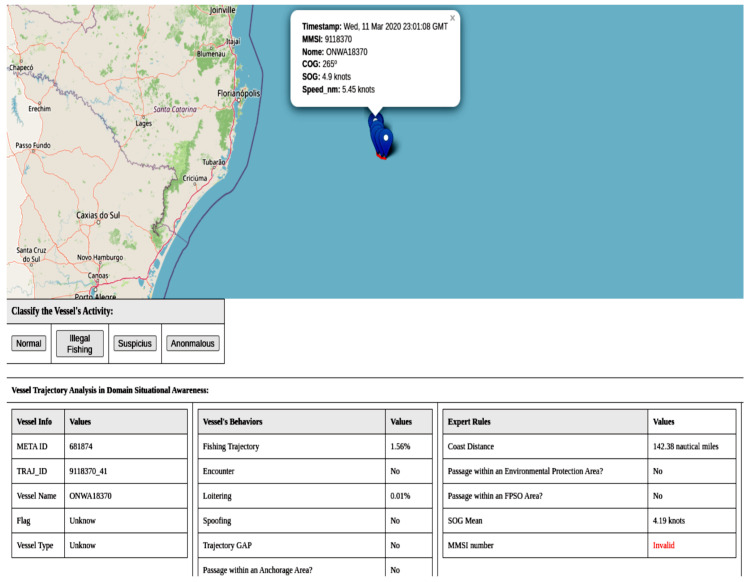
Experts evaluate trajectories through an interface at the impact assessment level. The user can have a situational awareness of a trajectory with vessel information, navigation behaviors presented by the trajectory, and whether the trajectory triggers the rules created by the expert.

The query strategy used to select the most informative data in active learning is *uncertainty sampling*. In this strategy, the model selects the samples about which it is most uncertain. The model then presents the trajectories to the user for labeling. Figure 9 shows an example of the user interface. At the impact assessment level, experts evaluate the trajectories through an interface. The user can have situational awareness of the trajectory with information about the vessel, navigation behaviors presented by the trajectory, and whether the trajectory triggers the expert-created rules.

The model updates itself after each labeling by the user. After a sufficient number of classifications (described in the Section 2.8), the model can infer activities for each received trajectory. Thus, the expert can now work only with trajectories that generate predictions of interest, such as illegal fishing and suspicious activities. In the next section, we will address this topic.

#### 2.7.5. Decision Support

This level is responsible for using the processed information to provide insights and support decision-making. The information obtained from the previous levels is integrated to support decision-making. First, based on the expert’s responses, we can calculate the performance of the framework’s predictions using precision, recall, and F1-score metrics. Additionally, with the trained metamodel, we can generate predictions for all unlabeled trajectories and present them on a map for the expert to evaluate by activity. This will save the expert time by focusing only on activities of interest.

This level has the capability to generate supplementary reports that improve situational awareness in the maritime area. For instance, specific information includes the mapping of vessel interactions, the identification of spots in which ships commonly anchor, and the identification of areas where AIS spoofing occurs regularly, among other situations.

### 2.8. Case Study

To test our framework, we used a practical case in Brazil’s EEZ, which covers a total of 3.6 million square kilometers. This maritime area includes complex and extensive navigation routes that connect various ports. For the tests, we used a dataset with AIS information collected from January 2019 to December 2020. First, it was necessary to clean this dataset. To do this, we used the following criteria. To remove missing or invalid data, consider that a trajectory must contain at least five points, the average trajectory speed must be between 1 and 50 knots, and the trajectories must be within Brazil’s EEZ. Using these criteria, we obtained 361,229 vessel trajectories, considering that each trajectory can contain a different number of points.

Due to the difficulty of finding datasets with the types of illegal activities we are trying to classify, we needed to create synthetic data to train our framework. For this, we used fishing trajectories found in the GFW dataset [57] and vessel encounters found in the MarineCadastre dataset [41]. We then translated the trajectories to random positions within the Brazilian EEZ. Thus, we could now train and test our framework. For training, we used the trajectories from the southeast region of the Brazilian coast, and, for testing, we used the trajectories from the south, southeast, north, and northeast regions of the coast. In the following sections, we will detail the construction of the synthetic dataset, the training and testing process of the framework, and the results.

#### Synthetic Dataset

A synthetic dataset is a dataset generated artificially rather than collected directly from the real world. These data can be created using algorithms and simulations that model the behavior of real systems or processes. The creation of synthetic datasets allows researchers and developers to test and validate algorithms in situations where real data are scarce, unavailable, or incomplete. In our case, we used a synthetic dataset due to the lack of labels for classifying illegal vessel activities. The available real data do not include enough information to accurately identify and label these activities, making it necessary to create a synthetic dataset that simulates these situations.

Our synthetic dataset was built in several steps:

- *Fishing trajectories from GFW randomly translated to the southeast coast of Brazil*: we used fishing trajectory data from the GFW dataset [57] and performed random translations of these trajectories to the southeast coast of Brazil. The regions where these trajectories were introduced were within the EEZ and within environmental areas of the southeast coast of Brazil;
- *Modification of the flag and type of the vessel*: to increase data heterogeneity, we altered the flag and type of some trajectories. The vessel’s flag can be *brazil*, *other*, or *unknown*. The vessel type can be *fishing*, *other*, or *unknown*;
- *Encounter trajectories from MarineCadastre translated to the southeast coast of Brazil*: vessel encounter data were obtained from the MarineCadastre dataset [41] and translated to the same southeast region, maintaining the same geographical coherence as the fishing data. The regions where these trajectories were inserted were within the EEZ and offshore exploration areas of the southeast region of Brazil;
- *Updating the AIS timestamp*: we updated the AIS timestamps to identify the synthetic data in the dataset;
- *Concatenation of synthetic data with real AIS data from the coast of Brazil (2019 to 2020)*: Finally, we combined the synthetic data with the real AIS data collected from the coast of Brazil from 2019 to 2020, creating a comprehensive and representative dataset.

Thus, we trained our framework using data from the southeast region and tested it across the entire coast to detect some real situations. By creating a synthetic dataset, we could train and validate our framework in more varied and challenging conditions, thereby enhancing its accuracy and effectiveness in detecting suspicious activities.

### 2.9. Active Learning Training

To train our metamodel, we executed the first three levels of our framework (Figure 8) using the synthetic dataset. In these stages, we performed data preprocessing, model and rule fusion, and the preparation and correlation of data for presentation to the expert. The synthetic dataset generated in the southeast coast region of Brazil comprises roughly 2.5 million AIS points, which were subsequently reduced to 46,924 trajectories after preprocessing. During the training with the specialist, we just utilized data from the southeast coast, as this was the region where we inserted the synthetic data.

**Figure 10:**
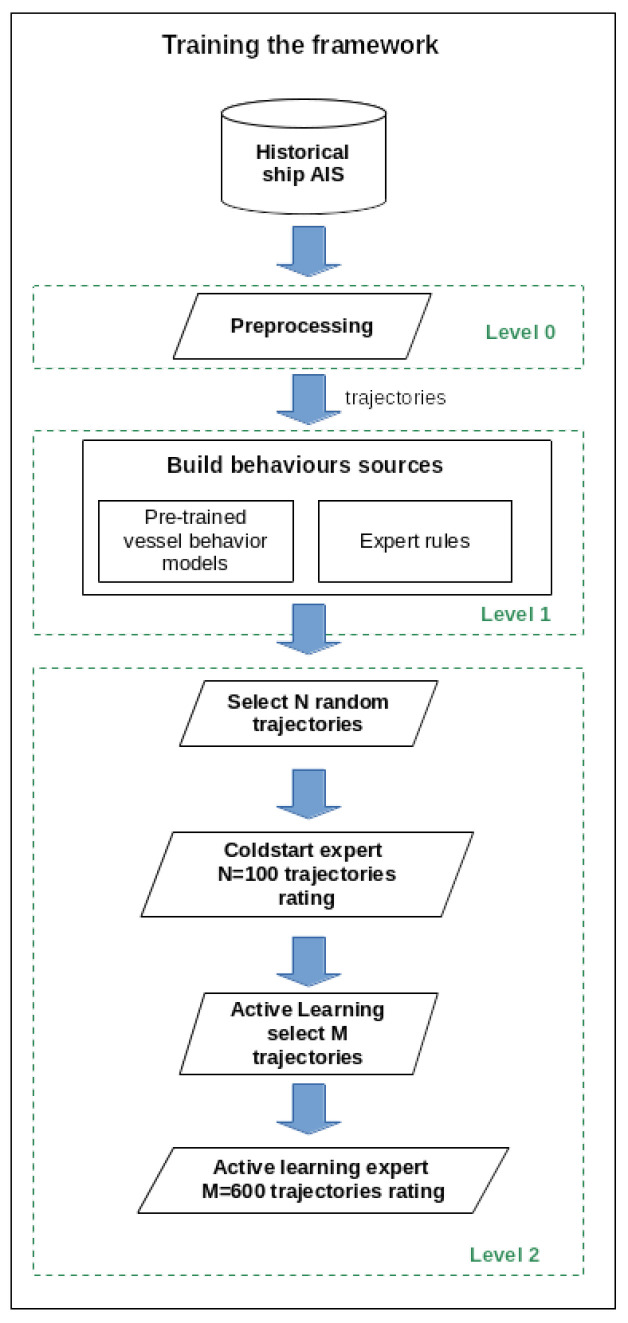
The active learning training process in the framework.

First, in active learning training, it is necessary to have some labeled data for the metamodel to be trained and infer new data. Therefore, a human operator initially evaluated 100 random trajectories, allowing the model’s cold start. Next, we trained the active learning metamodel, which suggests an additional 600 trajectories for the human operator to classify. During the active learning model training, we focused on the active selection of informative examples to prevent overfitting, ensuring that the model learned generalizable patterns. Additionally, during testing, we balanced the classes equally, which is important for preventing any bias and ensuring the robustness of the model. In Figure 10, we can see our framework’s entire training process.

In the case of the metamodel, since the dataset’s number of rows corresponds to the number of trajectories evaluated by the expert, the training of the metamodel takes only a few seconds. The majority of processing time was spent on preprocessing, building behaviors sources, and training the behaviors. Additionally, to find the optimal model parameters in model behaviors, it was necessary to run training and testing for each parameter tested. In our approach, we pre-trained the behaviors and saved them in a joblib format. During the framework execution, we simply loaded the models from the joblib files and applied them to the preprocessed trajectories. After training the framework, we carried out the testing process, which will be detailed in the next section.

### 2.10. Framework Testing

**Figure 11:**
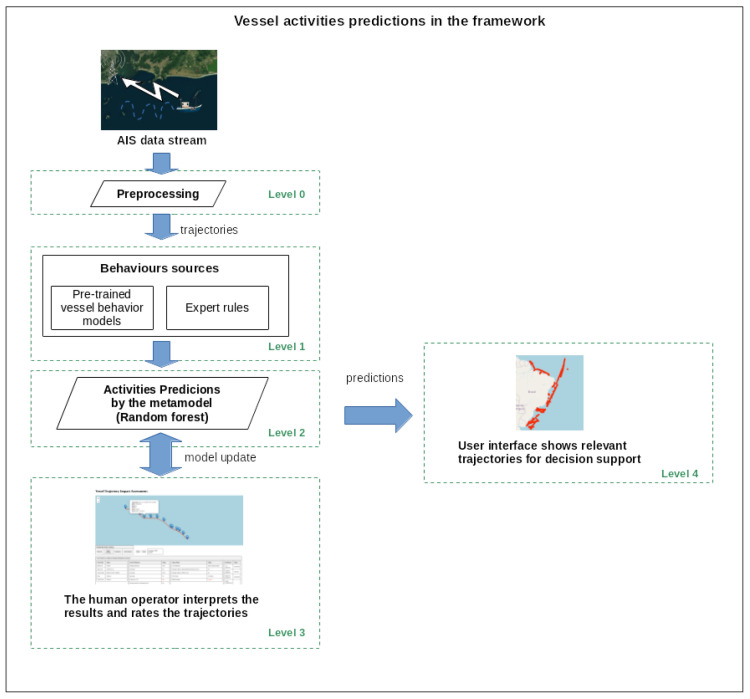
The maritime monitoring framework processing trajectories after training. Online data stream processing can make use of this procedure.

After training the framework, all other trajectories not used in the training dataset could be inferred, including the trajectories from the southern, northeastern, and northern coasts. To do this, these trajectories first needed to go through levels 0 to 2 of the framework, as shown in Figure 11. Then, for our validation test, 100 trajectories from each class (normal, illegal fishing, suspicious activity, and anomalous activity) inferred by the framework were selected and presented to the expert.

During the testing process, the expert evaluated a total of 400 trajectories, analyzing the behavior outputs and rules of each trajectory, and finally assigning their classification. In Figure 11, at Level 3, this evaluation process can be seen. Thus, based on the metamodel’s activity inference and the expert’s responses, we could now evaluate the performance of our framework.

To better understand the predictions of our metamodel, we used the SHAP (SHapley Additive exPlanations) tool. SHAP is a machine learning model interpretation tool based on Shapley values, which originate from game theory [58]. Shapley values provide a fair way to attribute the contribution of each feature to a model’s prediction, considering all possible combinations of features. SHAP allows for both global interpretation, providing an overview of feature importance across the entire dataset, and local interpretation, explaining the prediction of a single data point. Shapley values help us to understand how much each feature contributes to a model’s prediction, ensuring consistency and intuitive interpretation of the results. In the next section, we will present and analyze the results in more detail.

## 3. Results

As previously stated, the results obtained here were the result of a manual evaluation of each of the 400 trajectories inferred by the framework. We selected 100 random trajectories for each inferred activity class. To measure the framework’s performance, we used the metrics of *precision*, *recall*, *f1-score*, and *support*. Each metric can be defined as follows:

- **Precision**: this is the proportion of true positives among the instances that are classified as positive. It measures the accuracy of the model’s positive predictions.
- **Recall**: this is the proportion of true positives among all instances that are actually positive. It measures the model’s ability to find all positive instances.
- **F1-score**: this is the harmonic mean of *precision* and *recall*, providing a single metric that balances both considerations. It is especially useful when there is an imbalance between classes.
- **Support**: this refers to the number of actual occurrences for each class in the dataset. It is a measure that indicates how many instances are present for each class.

Additionally, **macro** and **weighted** averages were calculated:

- **Macro avg**: calculates the average of the metrics for all classes, treating each class equally, regardless of the number of instances.
- **Weighted avg**: calculates the average of the metrics for all classes, weighted by the number of instances in each class.

**Figure 12:**
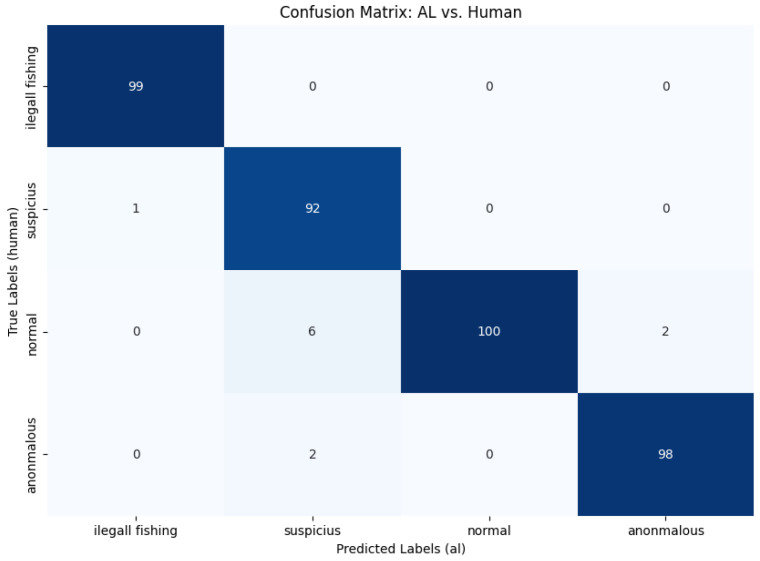
Confusion matrix of the predictions of the metamodel.

In Table 2, we can observe the performance of the four classes, highlighting that our framework achieved the best performance in detecting normal activities, with a performance of 100%, and illegal fishing activities, with a performance of 99%. In Figure 12, we can analyze the confusion matrix of the classes. From the matrix, we can observe that the framework, in some situations, incorrectly inferred the *suspicious* class instead of *normal* or *anomalous*, causing the model’s precision for this class to decrease. We also calculated the *macro* and *weighted* averages. As shown in Table 2, even with slightly different *support* in some classes, the averages were practically 97%.

**Figure 13:**
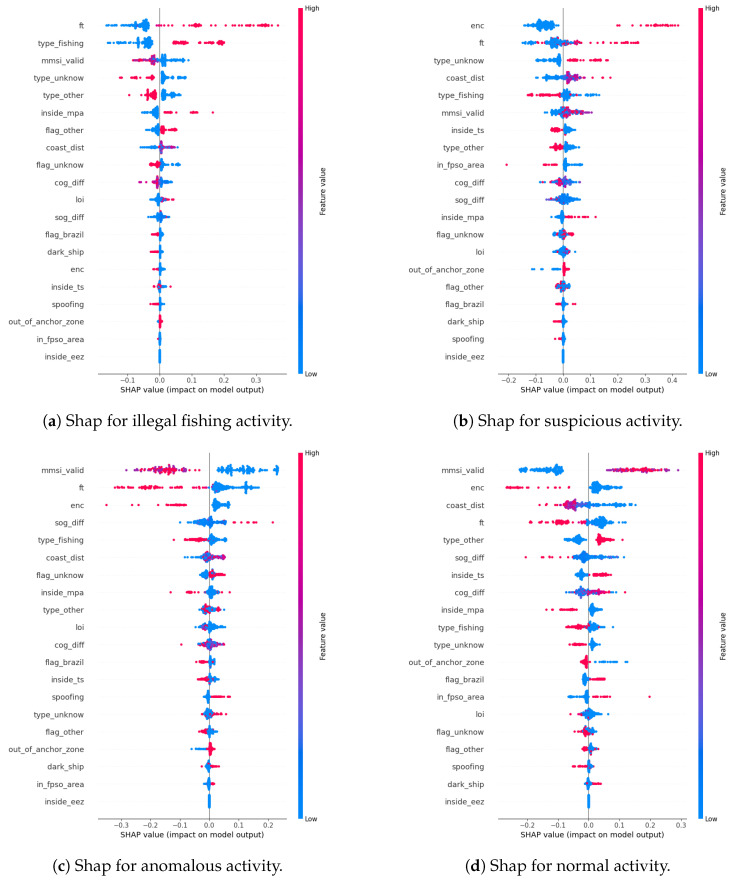
This figure shows the SHAP graphics for the metamodel predictions. The SHAP shows the contributions of each dimension to the class prediction.

In Figure 13, we have the plots for the four activities, where the *y*-axis shows the attributes used in the model and the *x*-axis shows how much each attribute impacted the prediction. Positive SHAP values (in red) indicate that the variable contributes to predicting the positive class, while negative values (in blue) indicate contribution to the negative class. The absolute size of the SHAP value indicates the magnitude of the contribution.

In Figure 13a, we can observe how the dimension values behave when inferring the illegal fishing class in our metamodel. In this graph, we can see that the most decisive dimension is *ft* (fishing trajectory), which is the output of the model that detects fishing behavior. In this case, the higher the *ft* value is, the greater is the impact on the model to infer illegal fishing. The same applies to the *type_fishing* dimension, which indicates whether the vessel is a fishing vessel or not. Since we used synthetic trajectories, the impactful dimensions are very noticeable due to the well-defined pattern established with the synthetic data, which may have facilitated the detection of these simpler cases by the model.

Regarding Figure 13b, we have the dimension values when the metamodel infers the suspicious activity class. In this case, we can see that the most impactful dimensions are *enc* (encounters) and *ft*. In this situation, if an encounter is detected, it is highly suspicious. The same happens with the fishing trajectory (*ft*). However, we can see that the *type_unknown* and *coast_distance* dimensions also have a strong influence on the result. In the case of a vessel with an unknown type (*type_unknown*), presenting a fishing trajectory (*ft*) within a marine protected area (*inside_mpa*), the metamodel considers it a suspicious activity. If *type_fishing* in this case were 1, it would be considered illegal fishing activity. Therefore, in this case, the higher the *type_fishing* value is, the lower the impact is for it to be classified as suspicious. For an encounter between vessels (*enc*) to be considered suspicious, the location must be outside an oil exploration area and more than 12 NM from the coast, meaning *in_fpso_area* must be 0 and *inside_ts* must be 0.

In Figure 13c, we have a case of activity classified as anomalous. In this case, we see from the three most impactful dimensions that, to be classified as an anomalous activity, the MMSI (*mmsi_valid* = 0) must be invalid, it cannot be a fishing trajectory (*ft* close to zero), and there should be no encounters (*enc* = 0). We can also observe that when *spoofing* = 1 and *out_of_anchor_zone* = 1, we have a well-defined distribution to be inferred as suspicious activity.

Finally, in Figure 13d, we can highlight the most impactful dimensions for classifying normal activity. The dimensions *mmsi_valid* greater than zero, *enc* = 0, *ft* close to zero, vessel type (*type_other*) different from fishing, and within the territorial sea (*inside_ts* = 1) influence the metamodel to classify the activity as normal. Thus, the model was able to accurately capture the characteristics of each activity that was initially only within the expert’s knowledge.

In a real-world scenario where we applied the framework to actual AIS data, we did not identify any real instances of illegal fishing; such cases were only detected in the synthetic trajectories. On the other hand, in the case of suspicious activities, we found real situations of suspicious encounters between vessels in the EEZ that could be trafficking activities, for example. We also discovered instances of fishing vessels operating within marine protected areas, potentially engaging in fishing activities for which our fishing trajectory detection model lacked training. In the future, we can incorporate additional behaviors or rules into the framework to classify activities more precisely, not just as suspicious.

In the case of anomalous activities, it is necessary to analyze repeating anomaly patterns and investigate the reasons. For example, in the case of an invalid MMSI, many smaller vessels use AIS transponders without registration with the competent authority and assign any number to the MMSI. This could be understood as a small vessel moving away from the coast to fish, for example. In the worst-case scenario, this same vessel may not want to identify itself as committing an illegal activity. Therefore, we need to study these anomaly patterns and incorporate other forms of behavior detection into the framework to identify such situations.

**Figure 14:**
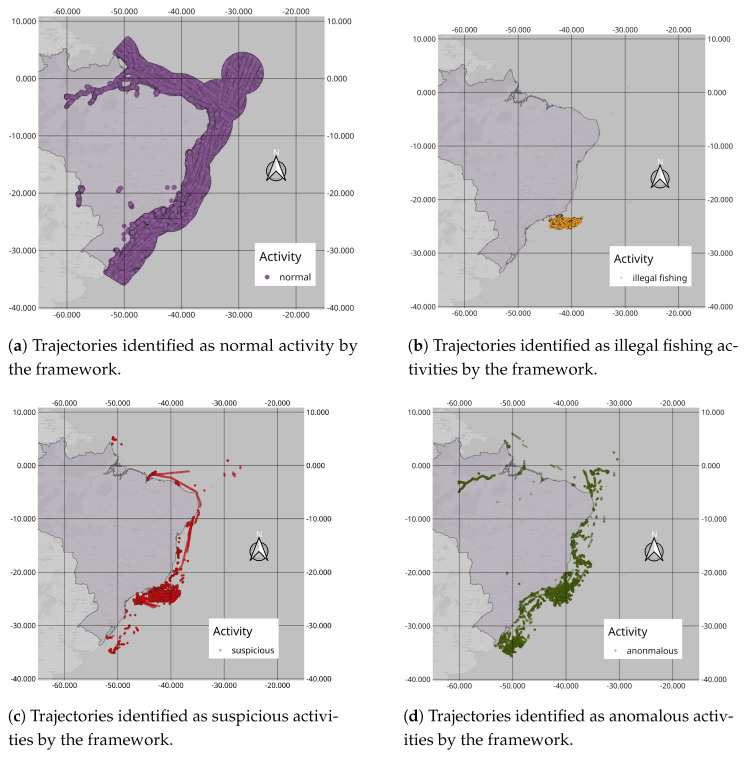
All trajectories inferred by the framework plotted on the map.

In Figure 14, we can see all the trajectories inferred by the framework and plotted on the map. In Figure 14a, we can see the trajectories considered normal activities, in Figure 14b those considered illegal fishing, in Figure 14c those considered suspicious activities, and in Figure 14d the activities inferred as anomalous. In Figure 14a, we observe the large number of trajectories, representing about 97% of the total trajectories, occupying almost the entire EEZ space. We also note that there are trajectories in rivers that are not included in our study.

In yellow, in Figure 14b, we can see that the trajectories considered illegal fishing are mainly concentrated in the southeast region. Indeed, all of them are synthetic trajectories, and the framework did not explicitly indicate any real illegal fishing activities based on the dataset used. In green, in Figure 14d, many trajectories in rivers are considered anomalous, as they were not part of our study and were not used in our training. Additionally, there are anomalous trajectories along the entire coast, mainly in the southeast and south regions. In some cases, navigation aid buoys were mistaken for vessels, either being transported with AIS on or in fixed locations.

In red, in Figure 14c, we can see that most of these trajectories are concentrated in the southeast region, exactly where we inserted the synthetic vessel encounter trajectories. However, the framework also identified other real dataset trajectories as potentially suspicious activities, as observed in other coastal regions.

**Figure 15:**
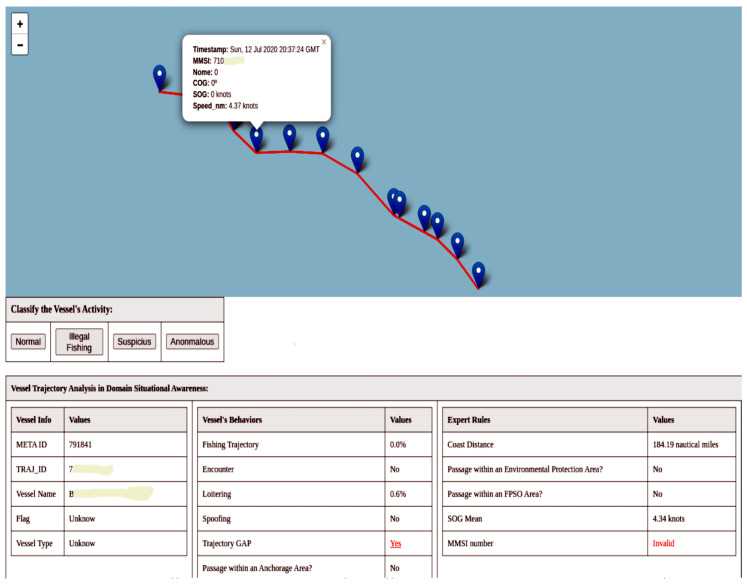
The image presents a real-world example of an anomalous activity that the framework has identified. An unidentified vessel with trajectory gaps and a sinuous path close to EEZ boundaries.

During the manual evaluations in the tests, we identified some real-world scenarios that warrant highlighting as examples. In Figure 15, we have a trajectory that our framework classified as anomalous. In the image, it is possible to observe the model outputs and the expert rules, where the vessel has an invalid MMSI, an unknown flag and type, a gap in its trajectory, and, visually, a sinuous trajectory with an average speed of 4.34 knots. Additionally, the vessel is close to 200 nautical miles, which is the EEZ limit. These anomalous behaviors alone cannot tell us what the vessel is trying to do, only that it is unusual.

**Figure 16:**
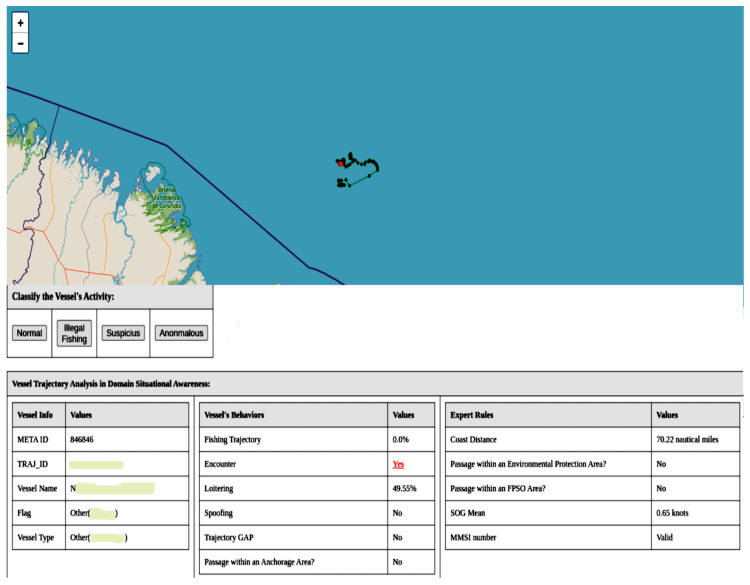
The image shows suspicious activity detected by the framework. The vessel represented by the green points is encountering the vessel represented by the red points at 70 NM off the coast.

In Figure 16, we have an example of suspicious activity classified by our framework, where two vessels meet approximately 70 NM from the coast. Vessel encounters are not illegal, but they can be an indication of illicit activity. To reduce false positives in activity detection, it is necessary to create models that detect behaviors, allowing for a better understanding of the situation. For instance, oil platforms require support vessels to transport oil, personnel, and materials. Therefore, encounters between vessels in these regions are common. The region where the encounter occurred, as shown in Figure 16, does not have oil exploration areas. Thus, the vessel had another reason for the encounter aside from offshore activity. Therefore, the specialist needs to pay closer attention to these cases.

Observing the SHAP graph of the suspicious activity class (Figure 13b), we see that, when an encounter between vessels is detected, far from the coast and outside an FPSO area, there is a high probability of predicting the suspicious activity class, due to these dimensions having a greater impact. These rules were implicitly inserted by the specialist during the active learning training. This is a significant advantage of the framework, which transforms the specialist’s tacit knowledge into explicit knowledge through the trained models.

**Figure 17:**
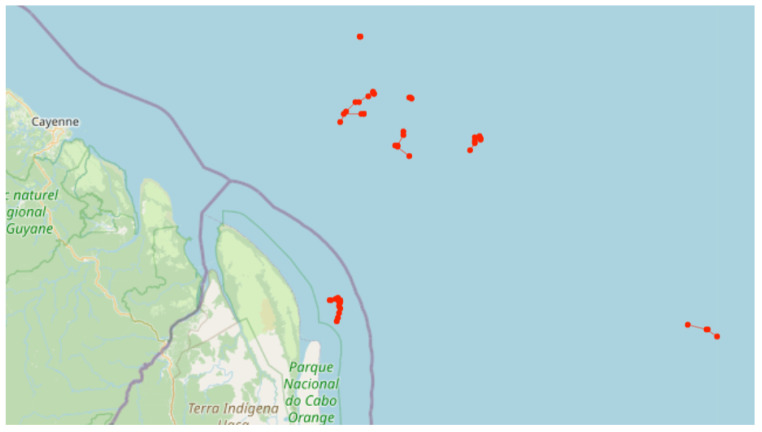
The figure shows trajectories detected as suspicious by the framework. The red points represent the trajectories of foreign fishing vessels along the northern coast of Brazil.

In northern Brazil, the framework identified suspicious activities involving the trajectories of fishing vessels from a foreign country. Although the model did not classify these trajectories as fishing, the vessels exhibited an average speed of 2 knots and sinuous trajectories, characteristics that typically occur in some fishing situations. The lack of training with this type of fishing may have led to a false negative for fishing in the fishing trajectory detection model. However, even without detecting them as fishing, the framework classified the trajectories as suspicious, as shown in Figure 17. Due to the location and the fact that the vessels belong to the same country, these activities are noteworthy and need to be analyzed in more detail by the specialist.

**Figure 18:**
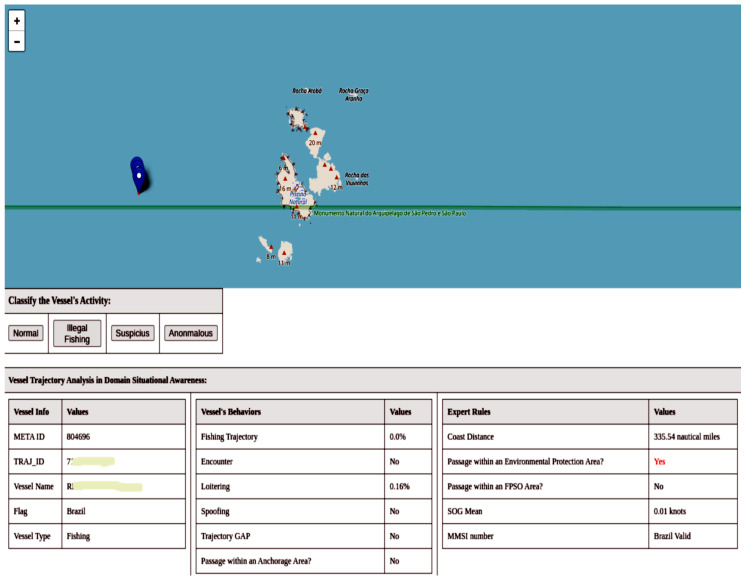
The figure shows a situation detected as suspicious activity by the framework. A vessel anchored within a marine protected area.

Another case worth highlighting, which our framework identified as suspicious, was a fishing vessel within the marine protected area of the São Pedro and São Paulo Archipelago. In Figure 18, we can see that the vessel is stationary; however, fishing is prohibited in this region. In preprocessing, we filtered for trajectories with speeds between 1 and 50 knots, which is the speed reported by the AIS. On the other hand, the average speed we calculated in the metamodel is the actual speed, based on the positions and the AIS point timestamps. The specialist must analyze this situation, as the vessel might be involved in another fishing modality where it stays stationary.

One of the main results of this research is the possibility of increasing human operator efficiency by eliminating the requirement to examine trajectories that are considered normal. We can perform a quantitative analysis to evaluate the workload required by human operators in this case study in the absence of our framework. In Table 3, we display the aggregate count of trajectories that have been analyzed by the framework.

In Table 3, we can see the number of activities inferred by our framework, where 1100 trajectories out of the total of 361,229 were used for training. Initially, in a maritime domain monitoring situation by specialists, we could avoid checking 350,780 trajectories that were considered normal activities, representing about 97% of the total trajectories. This makes sense, as a large portion of vessel trajectories are normal. Subsequently, specialists could prioritize more severe activities. In our case, we have the situation of illegal fishing.

## 4. Discussion

The results presented highlight the robustness and effectiveness of the proposed framework in classifying illegal activities in the maritime domain using vessel trajectory data. The high precision and recall, particularly in detecting illegal fishing and normal activities, demonstrate the model’s capability to accurately identify these activities. However, there were some limitations in classifying suspicious activities, which were sometimes confused with normal or anomalous activities, indicating the need for further refinement. This refinement could be achieved by increasing the number of trajectories in the training data and/or by adding other behaviors and/or rules that could better characterize these activities.

The SHAP analysis confirmed that the model’s predictions are consistent with expert knowledge, with features such as fishing trajectory and vessel type being key determinants in detecting illegal fishing. Identifying suspicious activities in real-world scenarios depends on detecting vessel encounters in unusual areas and operations in protected zones. The detection of these activities demonstrates the practical applicability of the framework. These results suggest that the framework could significantly reduce human operators’ workload by up to 97% by filtering out normal trajectories and allowing focus on critical cases. Despite being trained on synthetic data, the framework was able to accurately identify suspicious and anomalous activities in real-world scenarios, as evidenced in the preceding section.

However, the presence of false positives in detecting fishing trajectories showed that the curving paths of other vessels could be erroneously identified as fishing trajectories. This is especially noticeable in instances where cargo ship paths displayed fishing-like patterns with a probability exceeding 50%. When comparing the vessel’s data with other metamodel information, it was evident that the vessel was not involved in fishing activities. However, the curving and twisting path of the trajectory still caused the operator to be cautious about a possible abnormality. The training dataset used for fishing trajectories was given by GFW [57] and consists of data from large fishing vessels with particular features in their trajectories.

For comparison purposes, we could compare the performance of our results with existing literature. However, to the best of our knowledge, there are no prior studies that address our specific problem using the methodology we employed. Most works in the literature focus specifically on the detection of behaviors rather than on the overall activity of the vessel.

## 5. Conclusions

This work presents a framework capable of classifying vessel activities as illegal fishing, suspicious activity, anomalous activity, and normal activity. The framework combines the navigation behaviors presented by each trajectory with rules created based on the expert’s knowledge. We created a metamodel using the random forest model to perform the fusion. To address the problem of a lack of labels, we used active learning so that the expert could classify the trajectories with the highest probability of improving the model’s performance. We also built our framework based on the JDL model, which consists of five levels.

To train the behavior detection models, we used the GFW [57] and MarineCadastre [41] datasets. To train and test our metamodel using active learning, we used a synthetic dataset created by translating fishing and encounter trajectories from the GFW and MarineCadastre datasets to a real dataset from the coast of Brazil. In the tests we conducted, we can highlight the best performance, which was in detecting normal activities with 100% accuracy, illegal fishing with 99% accuracy, and the worst performance, which was in detecting suspicious activities with 92% accuracy.

Throughout the tests, our framework, trained with synthetic datasets, successfully identified real cases of vessels involved in suspicious encounters, as well as foreign vessels seemingly fishing within Brazil’s Exclusive Economic Zone (EEZ). This demonstrates that our framework for detecting illegal vessel activities is both viable and flexible in terms of adding new behaviors and activities, and it significantly reduces the number of trajectories that require expert attention.

As future work, testing with larger datasets and adding new behaviors, rules, and activities could further assess the framework’s scalability. The specialization of suspicious activities could be refined into more specific activities, such as drug trafficking or water pollution. The specification of these activities can be achieved by adding new behaviors/rules that detect the characteristics of these activities. Therefore, this work makes a significant contribution to the field of maritime surveillance, offering a scalable and efficient solution for detecting illegal activities in the maritime domain.

## Figures and Tables

**Table 1:** Table of expert-created rules and their corresponding purposes.

Rule	Purpose
Validation of the vessel’s MMSI, both syntactically and semantically [47]	To determine if the vessel has an MMSI registered with the local maritime authorities.
A crawler searches the internet using the MMSI and identifies the name, flag, and vessel type. As a result, we can generate the following attributes: *flag_brazil, flag_other, flag_unknown, type_fishing, type_other, type_unknown*	Identify the expected behavior based on the type and flag of the vessel.
Passage through anchorage areas	Identify if the vessel anchors in areas where ships typically anchor.
Distance from the coast	Based on the distance from the coast, identify the laws to which the vessel may be subject and the expected behavior in those regions.
Passage through marine protected areas (MPAs)	Determine if the vessel exhibits fishing behavior within areas where fishing is prohibited.
Passage through FPSO areas	Identify if the offshore support vessels conduct a rendezvous within these regions.
Average speed of the trajectory	Conduct a comparison between the average speed calculated based on latitude and longitude positions and the speed reported by the AIS. Additionally, analyze the correlation between the vessel’s behavior and its speed.

**Table 2:** Performance metrics of the metamodel: precision, recall, and F1-score for each activity class.

Activity	Precision	Recall	F1-Score	Support
Illegal fishing	0.99	1.00	1.00	99
Suspicious	0.92	0.99	0.95	93
Normal	1.00	0.93	0.96	108
Anomalous	0.98	0.98	0.98	100
**Accuracy**			0.97	400
**Macro avg**	0.97	0.97	0.97	400
**Weighted avg**	0.97	0.97	0.97	400

**Table 3:** Trajectories numbers by activity prediction, used in training and total.

Activity Predicted	Trajectories
Illegal fishing	393
Suspicious	2884
Anomalous	6072
Normal	350,780
Used in training	1100
Total	361,229

## Data Availability

The original data presented in the study used to train navigation behaviors are openly available in https://globalfishingwatch.org/data-download/datasets/public-training-data-v1 (Global Fishing Watch) and https://marinecadastre.gov/data (MarineCadastre.gov), accessed on 21 August 2024. However, the AIS data from the Brazilian coast presented in this article are not readily available because the authors do not have permission to share.

## Abbreviations

The following abbreviations are used in this manuscript:

AIS	Automatic Identification System
MA	Maritime authority
JDL	Joint Directors of Laboratories Data Fusion Model
SHAP	SHapley Additive exPlanations
AL	Active learning
EEZ	Economic Exclusive Zone
MPA	Marine protected area
MDA	Maritime Domain Awareness
ABM	Automated Behaviour Monitoring
EMSA	European Maritime Safety Agency
GFW	Global Fishing Watching
RNN	Recurrent neural network
COG	Course over ground
SOG	Speed over ground
MMSI	Maritime Mobile Service Identity
VHF	Very high frequency
NM	Nautical miles
FPSO	Floating Production Storage and Offloading
ft	Fishing trajectory
loi	Loitering
enc	Encounter
ts	Territorial sea

## References

[1] Stopford M. (2009). Maritime Economics.

[2] Forum W.E. (2024). Worlds Busiest Ocean Shipping Routes. https://www.weforum.org/agenda/2024/02/worlds-busiest-ocean-shipping-routes-trade.

[3] Crimes M. (2022). MICA Center—Annual Report 2021. https://maritimescrimes.com/2022/01/10/mica-center-annual-report-2021/.

[4] (2024). Sharda. A General Overview of Maritime Domain Awareness (MDA). https://www.marineinsight.com/maritime-law/a-general-overview-of-maritime-domain-awareness-mda/.

[5] Wolsing K., Roepert L., Bauer J., Wehrle K. (2022). Anomaly Detection in Maritime AIS Tracks: A Review of Recent Approaches. J. Mar. Sci. Eng..

[6] Dogancay K., Tu Z., Ibal G. (2021). Research into vessel behaviour pattern recognition in the maritime domain: Past, present and future. Digit. Signal Process. Rev. J..

[7] Rong H., Teixeira A., Soares C.G. (2024). A framework for ship abnormal behaviour detection and classification using AIS data. Reliab. Eng. Syst. Saf..

[8] Shi Y., Long C., Yang X., Deng M. (2022). Abnormal Ship Behavior Detection Based on AIS Data. Appl. Sci..

[9] Fahn C.S., Ling J., Yeh M.Y., Huang P.Y., Wu M.L. (2019). Abnormal Maritime Activity Detection in Satellite Image Sequences Using Trajectory Features. Int. J. Future Comput. Commun..

[10] Zocholl M., Iphar C., Jousselme A.L., Ray C. Ontology-based approach for vessel activity recognition. Proceedings of the OCEANS 2021: San Diego—Porto.

[11] Wang Y., Liu J., Liu R.W., Liu Y., Yuan Z. (2023). Data-driven methods for detection of abnormal ship behavior: Progress and trends. Ocean Eng..

[12] Ferlansyah N.M., Suharjito (2020). A systematic literature review of vessel anomaly behavior detection methods based on Automatic Identification System (AIS) and another sensor fusion. Adv. Sci. Technol. Eng. Syst..

[13] Watson J.R., Woodill A.J. (2022). Detecting illegal maritime activities from anomalous multiscale fleet behaviours. Fish Fish..

[14] European Maritime Safety Agency (EMSA) https://www.emsa.europa.eu.

[15] Androjna A., Perkovic M., Pavic I., Miskovic J. (2021). Ais data vulnerability indicated by a spoofing case-study. Appl. Sci..

[16] Androjna A., Perkovič M. (2021). Impact of Spoofing of Navigation Systems on Maritime Situational Awareness. Trans. Marit. Sci..

[17] Kontopoulos I., Spiliopoulos G., Zissis D., Chatzikokolakis K., Artikis A. Countering Real-Time Stream Poisoning: An Architecture for Detecting Vessel Spoofing in Streams of AIS Data. Proceedings of the 2018 IEEE 16th Intl Conf on Dependable, Autonomic and Secure Computing, 16th Intl Conf on Pervasive Intelligence and Computing, 4th Intl Conf on Big Data Intelligence and Computing and Cyber Science and Technology Congress(DASC/PiCom/DataCom/CyberSciTech).

[18] Kelly P. (2022). A novel technique to identify AIS transmissions from vessels which attempt to obscure their position by switching their AIS transponder from normal transmit power mode to low transmit power mode. Expert Syst. Appl..

[19] D’Afflisio E., Braca P., Willett P. (2021). Malicious AIS Spoofing and Abnormal Stealth Deviations: A Comprehensive Statistical Framework for Maritime Anomaly Detection. IEEE Trans. Aerosp. Electron. Syst..

[20] Shahir H.Y., Glasser U., Shahir A.Y., Wehn H. Maritime Situation Analysis Framework: Vessel Interaction Classification and Anomaly Detection. Proceedings of the 2015 IEEE International Conference on Big Data (Big Data).

[21] Matossian M., Laurila P., Blanchet C. (2020). Detecting dark vessels: Radar satellite-based monitoring of illegal activities at sea. Sea Technol..

[22] Uney M., Millefiori L.M., Braca P. Prediction of Rendezvous in Maritime Situational Awareness. Proceedings of the 2018 21st International Conference on Information Fusion (FUSION).

[23] Sharma A., Shekhar S. (2022). Analyzing Trajectory Gaps to Find Possible Rendezvous Region. ACM Trans. Intell. Syst. Technol..

[24] Niemeyer G. (2008). Geohash. http://geohash.org.

[25] Uber Technologies, Inc. (2018). H3: A Hexagonal Hierarchical Spatial Index. https://h3geo.org.

[26] Zhang T., Zhao S., Cheng B., Chen J. (2020). Detection of AIS closing behavior and MMSI spoofing behavior of ships based on spatiotemporal data. Remote Sens..

[27] Nikolic D., Stojkovic N., Popovic Z., Tosic N., Lekic N., Stankovic Z., Doncov N. (2019). Maritime over the horizon sensor integration: HFSWR data fusion algorithm. Remote Sens..

[28] Mazzarella F., Vespe M., Alessandrini A., Tarchi D., Aulicino G., Vollero A. (2017). A novel anomaly detection approach to identify intentional AIS on-off switching. Expert Syst. Appl..

[29] Kontopoulos I., Chatzikokolakis K., Zissis D., Tserpes K., Spiliopoulos G. (2020). Real-time maritime anomaly detection: Detecting intentional AIS switch-off. Int. J. Big Data Intell..

[30] Kontopoulos I., Makris A., Tserpes K. (2021). A deep learning streaming methodology for trajectory classification. ISPRS Int. J. Geo-Inf..

[31] Arasteh S., Tayebi M.A., Zohrevand Z., Glässer U., Shahir A.Y., Saeedi P., Wehn H. (2020). Fishing Vessels Activity Detection from Longitudinal AIS Data. Assoc. Comput. Mach..

[32] Zhang Z., Huang L., Peng X., Wen Y., Song L. (2022). Loitering behavior detection and classification of vessel movements based on trajectory shape and Convolutional Neural Networks. Ocean Eng..

[33] Ferreira M.D., Spadon G., Soares A., Matwin S. (2022). A Semi-Supervised Methodology for Fishing Activity Detection Using the Geometry behind the Trajectory of Multiple Vessels. Sensors.

[34] Zhao L., Shi G. (2019). Maritime Anomaly Detection using Densitybased Clustering and Recurrent Neural Network. J. Navig..

[35] Pedroche D.S., Amigo D., García J., Molina J.M. (2020). Architecture for trajectory-based fishing ship classification with AIS data. Sensors.

[36] Do Nascimento V.D., Alves T.A.O., Dutra D.L.C., Kundu S. A Comparative Study of Fishing Activity Detection Approaches in Maritime Surveillance. Proceedings of the 2023 Congress in Computer Science, Computer Engineering, & Applied Computing (CSCE).

[37] Watch G.F. (2024). Global Fishing Watch Fishing Effort Dataset. https://globalfishingwatch.org/datasets-and-code/fishing-effort/.

[38] Li W., Zhang D., Sun M., Yin Y., Shen Y. Loitering Detection Based on Trajectory Analysis. Proceedings of the 2015 8th International Conference on Intelligent Computation Technology and Automation (ICICTA).

[39] Lu R., Yang H., Zhu J., Wu S., Wang J., Bull D. Hierarchical Video Summarization with Loitering Indication. Proceedings of the 2015 Visual Communications and Image Processing (VCIP).

[40] Patino L., Ferryman J. (2017). Loitering Behaviour Detection of Boats at Sea. IEEE Comput. Soc..

[41] Cadastre M. (2024). Marine Cadastre National Viewer Datasets. https://marinecadastre.gov/data/.

[42] Sidibé A., Shu G. (2017). Study of automatic anomalous behaviour detection techniques for maritime vessels. J. Navig..

[43] Pan X., Wang H., Cheng X., Peng X., He Y. (2020). Online detection of anomaly behaviors based on multidimensional trajectories. Inf. Fusion.

[44] Laxhammar R., Falkman G. Conformal Prediction for Distribution-Independent Anomaly Detection in Streaming Vessel Data. Proceedings of the First International Workshop on Novel Data Stream Pattern Mining Techniques (StreamKDD ’10).

[45] Lu N., Liang M., Yang L., Wang Y., Xiong N., Liu R.W. Shape-Based Vessel Trajectory Similarity Computing and Clustering: A Brief Review. Proceedings of the 2020 5th IEEE International Conference on Big Data Analytics (ICBDA).

[46] Weintrit A. (2013). Marine Navigation and Safety of Sea Transportation: Navigational Problems.

[47] FCC (2024). Maritime Mobile Service Identities. https://www.fcc.gov/wireless/bureau-divisions/mobility-division/maritime-mobile/ship-radio-stations/maritime-mobile.

[48] de Faria J.A.P.M. (2012). A consciência situacional marítima (CSM) e a Marinha do Brasil. Nav. War Coll. J. Rev. Esc. Guerra Nav..

[49] Amazonia Azul. https://www.marinha.mil.br/delareis/?q=amazoniazul.

[50] Illegal, Unreported, and Unregulated Fishing. https://www.fisheries.noaa.gov/national/international-affairs/illegal-unreported-and-unregulated-fishing.

[51] Hastie T., Tibshirani R., Friedman J. (2009). The Elements of Statistical Learning: Data Mining, Inference, and Prediction.

[52] Zhou Z.H. (2012). Ensemble Methods: Foundations and Algorithms.

[53] Zhu X., Goldberg A.B. (2009). Introduction to Semi-Supervised Learning.

[54] Hall D.L., Llinas J. (1997). An introduction to multisensor data fusion. Proc. IEEE.

[55] Team G.D. (2018). GeoPandas: Python tools for geographic data. J. Open Source Softw..

[56] Graser A. (2021). MovingPandas: Efficient Structures for Movement Data in Python. https://github.com/anitagraser/movingpandas.

[57] Watch G.F. (2023). Global Fishing Watch Dataset. https://globalfishingwatch.org/data-download/datasets/public-training-data-v1.

[58] Lundberg S.M., Lee S.I. (2017). A Unified Approach to Interpreting Model Predictions. Proceedings of the 31st International Conference on Neural Information Processing Systems (NIPS’17).

